# Intelligent Genetic Decoding System Based on Nucleic Acid Isothermal Amplification for Non-Small Cell Lung Cancer Diagnosis

**DOI:** 10.3390/mi14030647

**Published:** 2023-03-12

**Authors:** Xiaonan Liu, Jiaxing Zhang, Kai Hua

**Affiliations:** 1 College of Forensic Medicine, Shanxi Medical University, Taiyuan 030001, China; 2 College of Life Sciences, Northwest University, Xi’an 710069, China

**Keywords:** loop-mediated isothermal amplification, epidermal growth factor receptor, L858R, non-small cell lung cancer

## Abstract

Non-small cell lung cancer (NSCLC) is a major cause of cancer-related deaths around the world. Targeting the sensitized epidermal growth factor receptor (*EGFR*) caused by gene mutation through the tyrosine kinase inhibitor is an effective therapeutic strategy for NSCLC. Hence, the individualized therapeutic strategy has highlighted the demand for a simple, fast, and intelligent strategy for the genetic decoding of *EGFR* to cater to the popularization of precision medicine. In this research, a one-pot assay for *EGFR* identification is established by combining a loop-mediated isothermal amplification and amplification refractory mutation system. By optimizing the component and condition of the nucleic acid amplification system, a sensitive and specific distinguishability is achieved for tracing target variant (60 copies, 0.1%) identification under a strong interferential background within 40 min. Moreover, complex operation and time-consuming data processing, as well as the aerosol contamination, are avoided owing to the whole process for intelligent genetic decoding being performed in a sealed tube. As a demonstration, L858R, the primary point mutation for the sensitization of *EGFR*, has been accurately decoded using this assay with highly heterogeneous cancerous tissue. In addition, this method can be easily extended for other genetic information decoding using a tailor-made primer set. Thus, we propose that this straightforward strategy may serve as a promising tool for NSCLC diagnosis in clinical practice.

## 1. Introduction

Lung cancer is a primary malignant tumor with a high incidence and mortality rate worldwide; non-small cell lung cancer (NSCLC) is the dominating subtype of lung cancer, which accounts for approximately 85% of these cases [1,2]. Along with the comprehensive molecular profiling of cancer-related genes, the insight into cancer treatment has been revolutionized and targeting the epidermal growth factor receptor (*EGFR*) through the tyrosine kinase inhibitor (TKI) has been regarded as an effective therapeutic strategy for NSCLC from a clinical oncology perspective [3,4]. However, the response of NSCLC patients to TKI therapy seriously depends on the mutant status in exons 18–21 of *EGFR*, which code for the tyrosine kinase domain [5,6]. Among these mutations, the L858R located in exon 21, which causes a leucine–arginine substitution at codon 858, is the primary point mutation, which comprises about 41% of all activating mutations that confer sensitivity to TKI therapy [5,7]. Hence, the L858R point mutation of *EGFR* is a crucial biomarker for decision making in the treatment of NSCLC to obtain the individualized therapeutic strategy for patients. Moreover, the mutant status of *EGFR* should be decoded as soon as possible to provide timely personalized therapy due to most NSCLC patients being diagnosed in the advanced or metastatic stages. Hence, the strategies for *EGFR* mutation identification should meet the requirement of the speediness and convenience for the point of care test (POCT), which is defined as the test being performed near the patient and the result potentially leading to a preferable therapy for the patient [8].

As *EGFR* mutation is highly responsive to TKI therapy in NSCLC treatment, *EGFR* mutation decoding has attracted extensive attention and a variety of strategies have been established. The therascreen *EGFR* RGQ PCR Kit (Qiagen Co., Ltd., Hilden, Germany) is developed based on real-time PCR for the detection of 21 *EGFR* mutations located in exons 18–21; this commercial kit has been approved as a companion diagnosis for NSCLC by the United States Food and Drug Administration [9,10]. In addition, massively paralleled DNA sequencing, which enables millions of target genes to be sequenced simultaneously, has been introduced into *EGFR* mutation decoding and screening [11,12]. However, due to the sophisticated equipment and time-consuming procedure, as well as the complex data analysis necessary with these methods, it is difficult to satisfy the demand for the speediness and convenience for POCT in clinical practice. Hence, a simple and fast strategy for *EGFR* mutation identification is still desired for providing timely genetic information in NSCLC personalized treatment.

Loop-mediated isothermal amplification (LAMP) is an excellent nucleic acid amplification technology under the isothermal condition that has been widely used in pathogen detection [13,14,15]. Compared with PCR, by employing more primers and higher primer concentration, LAMP exhibits higher sensitivity and specificity, as well as a higher amplification efficiency [13]. Furthermore, the expensive thermocycler is free for LAMP, which reduces the testing cost in medical institutions, especially in resource-constraint settings. Taking advantage of LAMP, some methods based on LAMP have been established for *EGFR* mutation decoding, including the LAMP-based melting curve [9,16], LAMP-based microfluidic device [17,18], and LAMP-based lateral flow device [19]. However, the melting curve process weakens the speediness of LAMP by lengthening the detection time and the fabrication of microfluidics and the lateral flow device increases the testing cost. Although these LAMP-based strategies are not popularized in clinical practice, LAMP still provides a simple, rapid, and low-cost approach for *EGFR* mutation identification.

In this research, by combining LAMP and amplification refractory mutation system (ARMS), a simple, fast, and low-cost strategy for intelligent *EGFR* decoding, named One-Pot-LAMP, is established, which can provide timely genetic information within 40 min for personalized NSCLC treatment. Moreover, the false positive result from aerosol contamination is avoided owing to the result being directly interpreted in a sealed tube during the whole process. In addition, further complex operations and time-consuming data analysis are avoided as well. The combination of speediness and convenience causes this assay to be a great candidate for NSCLC diagnosis in POCT scenarios.

## 2. Materials and Methods

### 2.1. Oligonucleotide

To establish One-Pot-LAMP, two primer sets for *EGFR* L858R decoding were designed using the Primer 5.0 software program (Primer-E Co., Ltd., Plymouth, UK). For convenience, mutant and wild type are noted as “M” and “WT”, respectively, in this research. The M primer set includes two M inner primers (FIP m and BIP-M) for recognizing the mutant variant and two outer primers (F3 and B3). The WT primer set includes two WT inner primers (FIP-WT and BIP-WT) for recognizing the wild-type variant, as well as two outer primers (F3 and B3). In addition, two primers were designed as well for plasmid construction and DNA sequencing. All the primers were synthesized, Invitrogen Biotechnology Co., Ltd. (Shanghai, China) (Table S1 and Figure S1).

### 2.2. Plasmid Construction

Two plasmids containing the sequences of wild-type or L858R mutation of *EGFR*, respectively, were constructed using a pMD19-T Vector Cloning Kit (Takara Co., Ltd., Dalian, China). Then, the recombinant plasmids were transformed into the *Escherichia coli* DH5α cells (Takara Co., Ltd.) and extracted using a TIANprep Mini Plasmid Kit (Tiangen Biotech Co., Ltd., Beijing, China). All the plasmids were confirmed through DNA sequencing by Beijing Genomic Institute (Beijing, China) and the concentrations were determined using a NanoDrop 2000 UV–Vis spectrophotometer (Thermo Fisher Scientific Co., Ltd., Wilmington, DE, USA).

### 2.3. Cell Culture and Clinical Sample

Two lung cancer cell lines containing L858R point mutation (NCI-H1975) and the wild-type variant (A549) of *EGFR* as the simulative positive and negative specimens, respectively, were purchased from Procell Life Science Co., Ltd. (Wuhan, China). The cell lines were cultured in the RPMI 1640 medium (HyClone Co., Ltd., Logan, UT, USA) with 10% fetal bovine serum (Gemini Bio Co., Ltd., West Sacramento, CA, USA) using a cell incubator (MCO-5AC, SANYO Electric Co., Ltd., Osaka, Japan) at 37 °C with 5% CO_2_. The cells at the logarithmic phase were detached using 0.25% trypsin-EDTA (Gibco Co., Ltd., Grand Island, NY, USA) for the subsequent study.

The cancerous tissues were surgically resected from 40 patients diagnosed with non-small cell lung cancer by the expert pathologist at the First Affiliated Hospital of Xi’an Jiaotong University (Xi’an, China) with informed consent. Then, all the cancerous tissues were formalin-fixed and paraffin-embedded for the subsequent study. This research was approved by the Ethics Committee of the College of Life Sciences, Northwest University (Xi’an, China), and all human-related protocols were performed in accordance with the Declaration of Helsinki.

### 2.4. Genomic DNA Purification

The genomic DNA from the cell lines and formalin-fixed and paraffin-embedded cancerous tissue were extracted using a TIANamp Genomic DNA Kit (Tiangen Biotech Co., Ltd.) and a TIANquick FFPE DNA Kit (Tiangen Biotech Co., Ltd.), respectively. The concentrations were further determined using a NanoDrop 2000 UV–Vis spectrophotometer.

### 2.5. One-Pot-LAMP Design

For the *EGFR* L858R decoding using this method, two paralleled amplification reactions (M tube added with M primer set and WT tube added with a WT primer set) were simultaneously performed for each specimen determination by recognizing the mutant variant (M reaction) and wild-type variant (WT reaction). For each amplification, a 25 μL reaction mixture containing 15 μL of Isothermal Master Mix (OptiGene Co., Ltd., West Sussex, UK), 0.2 µM of F3 and B3, 1.0 µM of FIP and BIP, and genomic DNA from the specimen was incubated at 63 °C for 40 min and the amplification kinetics were obtained in real time using a fluorometer (OptiGene Co., Ltd.). To achieve the best discrimination between the two variants for *EGFR* decoding, the amplification component was optimized with different inner primer concentrations, and the amplification condition was optimized using different incubation temperatures as well.

## 3. Results

### 3.1. One-Pot-LAMP Design

ToTo decode the L858R located in *EGFR*, a pair of inner primers were correspondingly tailor-made for recognizing the target variant through their DNA sequences. The point mutant nucleotide of L858R was located between the F1 and B1c regions and the two regions overlapped in this mutant nucleotide. Hence, the crucial nucleotide of the primer set for decoding *EGFR* L858R was designed at the 5′ terminus of the inner primer. The six regions located in *EGFR* were well-chosen for amplification with high specificity from the whole human genome. Moreover, to achieve the best discrimination between the two variants, an additional mismatched nucleotide was introduced at the 5′ penultimate of the inner primer according to the principle of ARMS [20].

**Figure 1:**
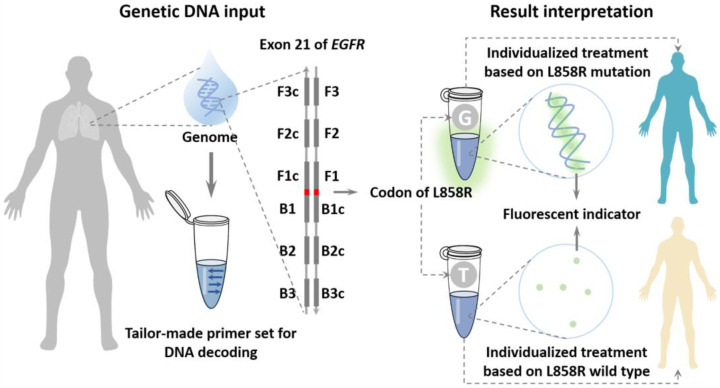
Schematic of One-Pot-LAMP for *EGFR* L858R decoding from genetic DNA input to result interpretation in a sealed tube.

The mutant status of *EGFR* L858R was determined by the two parallel LAMP reactions. Specifically, double-stem-loop structures were formed through strand displacement in both reaction mixtures and served as the trigger for self-primed DNA synthesis. In the matched primer–variant tube, the double-stem-loop structure containing complementary nucleobase with the target variant would be formed, which resulted in exponential DNA amplification and the accumulation of stem-loop DNA with various stem lengths. However, the double-stem-loop structure containing a non-complementary nucleobase would be formed in the mismatched primer–variant tube, which failed to cause self-primed DNA synthesis. Therefore, the S-shaped amplification kinetic curve derived from the exponential DNA amplification would be observed only when the specimen contains the matched variant with the primer set (Figure 1).

**Figure 2:**
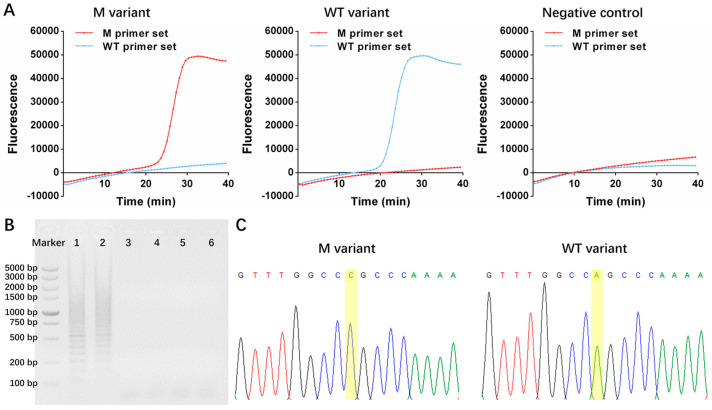
The amplification kinetic curve of One-Pot-LAMP with different variants (**A**) and detection result provided using electrophoresis (lane 1: M primer set with M variant, lane 2: WT primer set with WT variant, lane 3: M primer set with WT variant, lane 4: WT primer set with M variant, lane 5: M primer set with deionized water, and lane 6: WT primer set with deionized water) (**B**) and DNA sequencing (**C**).

The mutant status of *EGFR* L858R could be decoded according to the fluorescent signal derived from both amplification mixtures. For the mutant variant, a significant amplification signal could be observed in the M tube, while the wild-type variant led to an amplification signal only from the WT tube. Compared with the mismatched primer–variant pair, the matched primer–variant pair produced a significant S-shaped amplification kinetic curve, which facilitated determining the *EGFR* mutation status. For the negative control with deionized water as the template, the amplification kinetic curves were consistent with those from the mismatched primer–variant pair for both M and WT tubes (Figure 2A). Moreover, the amplification kinetic curve indicated that the accurate result could be provided within 40 min. As a reference, the amplicon from the LAMP was also analyzed using electrophoresis. The typical ladder-like pattern was observed from the matched primer–variant pair while the blank lane was observed from the mismatched primer–variant pair, which indicated that the S-shaped amplification kinetic curve was not caused by spurious amplification (Figure 2B). In addition, DNA sequencing was also employed to determine and obtain a perfect correlation (Figure 2C).

### 3.2. One-Pot-LAMP Optimization

**Figure 3:**
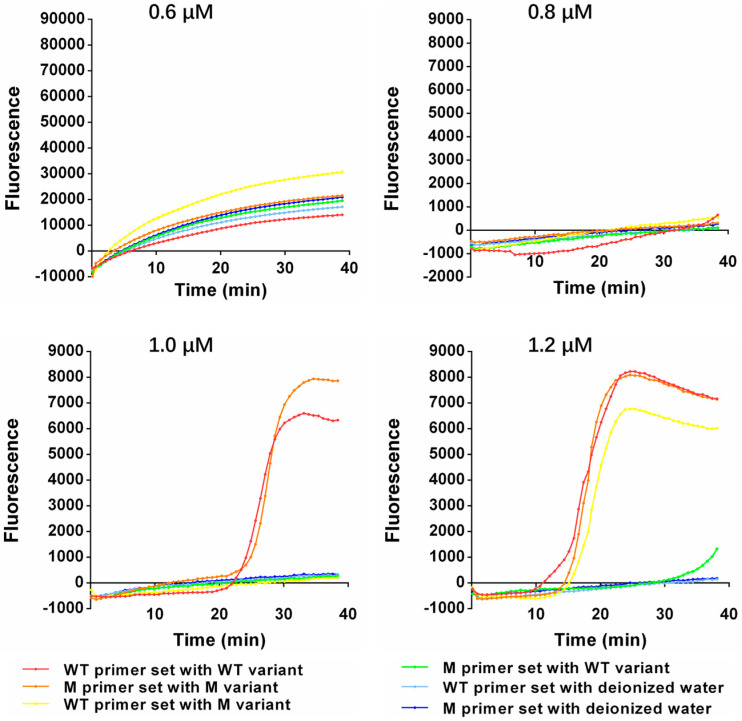
The amplification kinetic curve of One-Pot-LAMP with different concentrations of inner primer.

**Figure 4:**
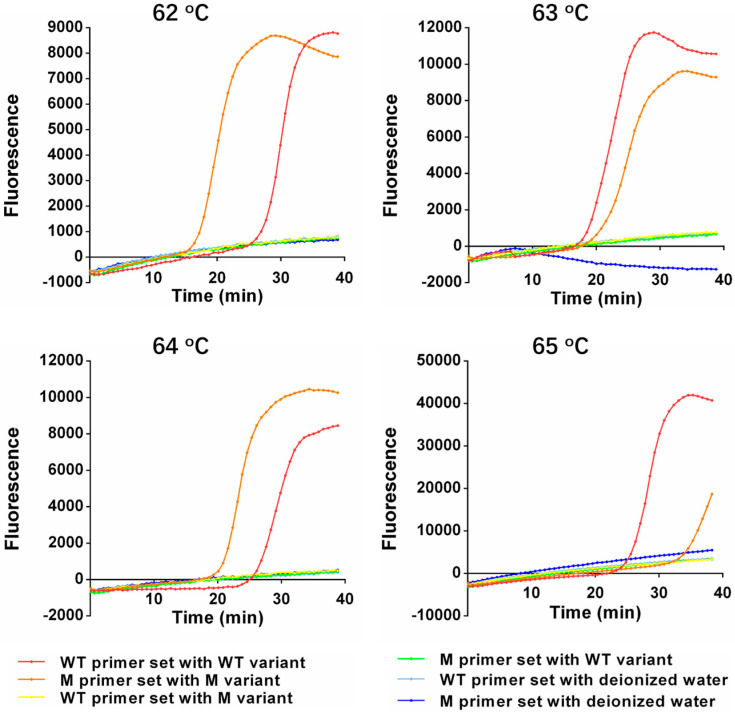
The amplification kinetic curve of One-Pot-LAMP under different incubation temperatures.

To decode *EGFR* L858R with high sensitivity and specificity as fast as possible, the performance of the One-Pot-LAMP was optimized with different inner primer concentrations and different incubation temperatures. The plasmids containing the sequences of the wild-type or L858R mutations of *EGFR*, respectively, were employed to perform the optimization. For the optimal primer concentration, the performance of this method with different final concentrations (0.6, 0.8, 1.0, and 1.2 μM) of the inner primer in the reaction mixture was evaluated. Among all the tested concentrations of the inner primer, 0.6 and 0.8 μM were too low to trigger the amplification within 40 min in both matched and mismatched primer–variant pairs. While a distinct amplification signal was observed in the matched primer–variant pair, and the amplification signal was not observed in the mismatched pair when the inner primer concentration rose to 1.0 μM, which allowed for a clear discrimination between the wild-type and L858R mutations of *EGFR*. However, when the concentration rose to 1.2 μM, further non-specific amplification was observed in the mismatched primer–variant pair. Hence, 1.0 μM was selected as the optimal concentration of the inner primer in this assay (Figure 3). Based on the optimal inner primer concentration, the performance of this method with different incubation temperatures (62, 63, 64, and 65 °C) was also evaluated. Among all the tested temperatures, a distinct amplification signal was only observed in the matched primer–variant pair. However, the amplification signals in the M reaction mixture were deferred at 62, 64, and 65 °C compared with 63 °C, while the amplification signals in the WT reaction mixture were similar at 63 and 64 °C but earlier than those at 65 °C. Thus, 63 °C was chosen as the optimal temperature in this assay (Figure 4).

### 3.3. One-Pot-LAMP Performance

**Figure 5:**
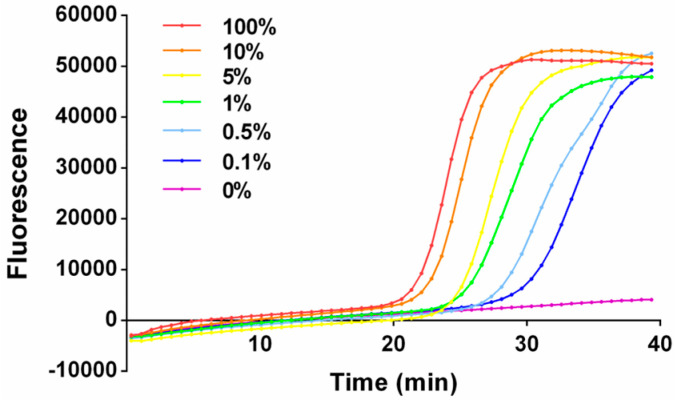
The amplification kinetic curve of One-Pot-LAMP with different proportions of target variant.

Subsequently, the performance of One-Pot-LAMP under the optimal condition was evaluated and plasmids were also employed as standard sequence control. Different plasmid mixtures containing 0% to 100% mutant plasmid diluted in wild-type plasmid with a final amount at 200 fg were prepared. The total plasmid mixture was added to the reaction mixture, respectively to evaluate the capacity of this method to recognize the target variant under interferential background. Distinct S-shaped amplification kinetic curves were observed for all test cases containing target variant within 40 min even when the target plasmid was as low as 0.1% (0.2 fg, approximately 60 copies). While no amplification signal was observed for the plasmid mixture containing no target variant, which indicated the high sensitivity and specificity of this method (Figure 5).

### 3.4. Cell Lines and Clinical Samples Analysis

**Figure 6:**
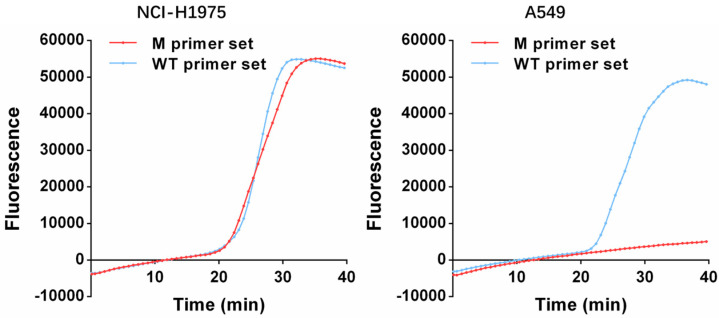
The amplification kinetic curve of One-Pot-LAMP with genomic DNA from cell lines.

Next, the performance of this method was further evaluated using the lung cancer cell lines and the *EGFR* L858R mutant statuses were identified using the 10 ng genomic DNA extracted from the NCI-H1975 and A549 cell lines, respectively. Significant amplification signals were observed from both M and WT reaction mixtures, which indicated that the *EGFR* L858R mutant status was a point mutation in NCI-H1975, while the S-shaped amplification kinetic curve was only observed from the WT reaction mixture, which indicated that the *EGFR* L858R mutant status was a wild-type variant in A549 (Figure 6). These results were consistent with those from the previous research [17,18]. Furthermore, the *EGFR* L858R mutant statuses of 40 cancerous tissues from 40 non-small cell lung cancer patients were also identified using this method. Among these real clinical specimens, 34 L858R mutation cases and 6 wild-type cases were observed. Meanwhile, the TaqMan-PCR *EGFR* Kit obtained from Life gene Co., Ltd. (Xi’an, China) was also employed to analyze these cancerous tissues for the double-blind trial. Compared to the results from the two methods, no discrepancy was observed, which indicated that this One-Pot-LAMP was able to accurately identify the *EGFR* L858R mutant status for clinical practice (Table 1).

## 4. Discussion

Lung cancer has been widely recognized as a seriously threatening tumor with a high incidence and mortality rate worldwide. NSCLC accounts for about 85% of all types of lung cancer [1,2]. In clinical practice, different genetic mutations of NSCLC in patients result in different therapeutic responses with the same drug [3,21]. Among these mutations, *EGFR* mutation has been found in approximately 35% of NSCLC patients in East Asia; the L858R located in the tyrosine kinase domain of *EGFR* is the most common point of mutation, accounting for about 41% of all *EGFR* mutations [7,22]. The mutant status of *EGFR* is highly responsible for the therapeutic effect of TKI, which is an effective therapeutic strategy for NSCLC [5,6]. Hence, individualized treatment is always desired by *EGFR* mutation decoding owing to the NSCLC patient survival rate being significantly improved by an early diagnosis [23]. For *EGFR* mutation decoding, LAMP is an excellent nucleic acid amplification technology that provides a simple, rapid, and intelligent approach; several LAMP-based methods have been developed for *EGFR* mutation detection [9,17,18,19]. However, the analysis of amplicons using LAMP lengthens the detection time and the fabrication of a micro device increases the testing cost. In this research, by combining LAMP and ARMS, the L858R point mutation of *EGFR* can be accurately identified through the optimal nucleic acid amplification system within 40 min, which is significantly simpler and faster than the methods mentioned above.

Cancerous tissue is highly heterogeneous and the genetic decoding method must be as sensitive as possible. For One-Pot-LAMP, by employing a tailor-made primer set targeting L858R mutation, high sensitivity and specificity are achieved to identify the trace of the target variant. As low as 0.1% (60 copies) of the target variants can be accurately decoded under a strong interferential background. Hence, the earlier diagnosis and individualized therapeutic strategy for NSCLC patients may be obtained for clinical practice. Moreover, this method is free of any expensive equipment and fluorescein-labeled oligonucleotide, which causes this method to be very low-cost and suitable for application in resource-limited medical institutions. In addition, time-consuming procedures and complex data analysis are avoided, which causes this method to be more convenient. Hence, the One-Pot-LAMP established here may serve as a promising tool for NSCLC diagnosis, therapy, and prognosis in POCT scenarios owing to its advantages of being simple, fast, and low-cost.

To achieve one-pot and intelligent *EGFR* decoding, the amplification signal of this nucleic acid system is employed for result interpretation. Hence, the primer set for the target variant is designed cautiously and the principle of ARMS was introduced to enhance the specificity. The formation of a double-stem-loop structure is a trigger for exponential DNA amplification. Hence, additional mismatched nucleotides at the 5′ penultimate of both FIP and BIP are designed, and the exponential DNA amplification only begins when the double-stem-loop structure contains a complementary nucleobase with the target variant. In addition, aerosol contamination, the major reason for false positive results in LAMP-based methods, was avoided, as the whole detection process is performed in a sealed tube.

## 5. Conclusions

In summary, One-Pot-LAMP, a simple, fast, and low-cost strategy for intelligent *EGFR* decoding, was established. It can provide timely genetic information within 40 min for personalized NSCLC treatment. One-Pot-LAMP not only achieves high sensitivity and specificity but can also be easily extended for other genetic information decoding using redesigned primer sets. The performance of One-Pot-LAMP was verified using real-world samples to determine the *EGFR* L858R mutation, which indicates the great potential of this straightforward strategy for NSCLC diagnosis in clinical practice.

## Figures and Tables

**Table 1:** Detection result of *EGFR* L858R with clinical sample.

One-Pot-LAMP	TaqMan-PCR *EGFR* Kit				Agreement
	Wild Type	L858R Mutation	Discrepant	Total	
Wild type	6	0	0	6	100%
L858R mutation	0	34	0	34	100%
Total	6	34	0	40	100%

## Data Availability

The data are available within this article.

## Supplementary Materials

The following supporting information can be downloaded at: https://www.mdpi.com/article/10.3390/mi14030647/s1, Table S1: Primer sequences for One-Pot-LAMP and plasmid construction. Figure S1: The amplification kinetic curve of One-Pot-LAMP with different concentrations of inner primer.
