# Supplementary material for: Intelligent Genetic Decoding System Based on Nucleic Acid Isothermal Amplification for Non-Small Cell Lung Cancer Diagnosis
Source: Micromachines (Basel). 2023 Mar 12;14(3):647. doi: 10.3390/mi14030647 (PMC10051770; doi:10.3390/mi14030647)
Supplement: Supplementary file 1 [file micromachines-14-00647-s001.zip › micromachines-2218263-supplementary.pdf]

Intelligent Genetic Decoding System Based on Nucleic Acid Isothermal Amplification for Non-Small Cell Lung Cancer Diagnosis

Xiaonan Liu[1,2,†,*], Jiaxing Zhang[2,†], Kai Hua[2]

[1] College of Forensic Medicine, Shanxi Medical University, Taiyuan, Shanxi, 030001, China.

[2] College of Life Sciences, Northwest University, Xi'an, Shaanxi, 710069, China.

[*] Corresponding Authors.
E-mail addresses: xiaonanliuvip@163.com.

[†] These authors contributed equally to this work.

Table S1. Primer sequences for One-Pot-LAMP and plasmid construction

Application	Primer	Sequence (5'-3')
One-Pot-LAMP	FIP-M	CACCCAAAATCTGTGATCTTGGCAGCCAGGAACGTACTG
	FIP-WT	AACCCAAAATCTGTGATCTTGGCAGCCAGGAACGTACTG
	BIP-M	GAGCCAAACTGCTGGGTGCGGATGCCTCCTTCTGCATGGTA
	BIP-WT	TAGCCAAACTGCTGGGTGCGGATGCCTCCTTCTGCATGGTA
	F3	CTTGGAGGACCGTCGCTT
	B3	ATGCTGGCTGACCTAAAGC
Plasmid construction	Forward	TTCTTCCCATGATGATCTGTCC
	Reverse	CCTCCCCTGCATGTGTTAAAC

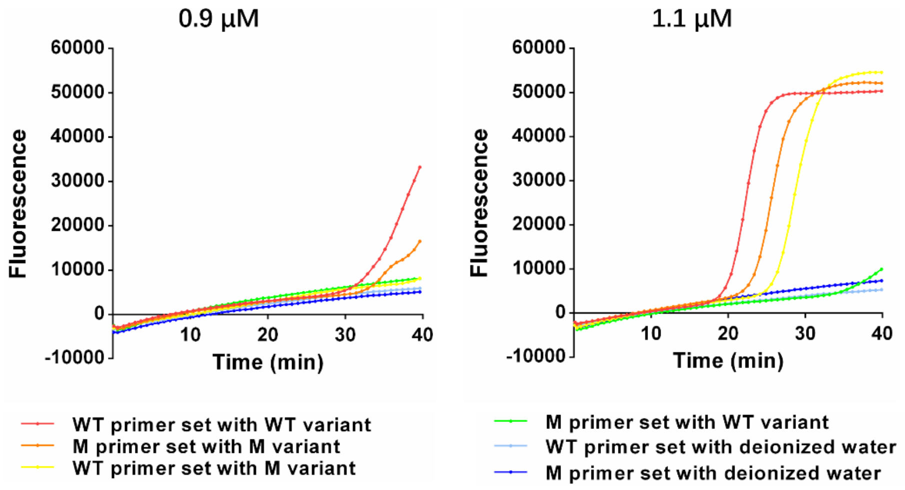

Figure S1. The amplification kinetic curve of One-Pot-LAMP with different concentrations of inner primer.
